# Isosamidin from *Peucedanum japonicum* Roots Prevents Methylglyoxal-Induced Glucotoxicity in Human Umbilical Vein Endothelial Cells via Suppression of ROS-Mediated Bax/Bcl-2

**DOI:** 10.3390/antiox9060531

**Published:** 2020-06-17

**Authors:** Moon Ho Do, Jae Hyuk Lee, Jongmin Ahn, Min Jee Hong, Jinwoong Kim, Sun Yeou Kim

**Affiliations:** 1 Korea Food Research Institute, 245, Nongsaengmyeong-ro, Iseo-myeon, Wanju-gun, Jeollabuk-do 55365, Korea; Do.Moon-ho@kfri.re.kr; 2 College of Pharmacy, Gachon University, #191, Hambakmoero, Yeonsu-gu, Incheon 21936, Korea; wogur5378@gc.gachon.ac.kr; 3 College of Pharmacy and Research Institute of Pharmaceutical Sciences, Seoul National University, 1 Gwanak-ro, Gwanak-gu, Seoul 08826, Korea; jm212224@snu.ac.kr (J.A.); everpolaris@snu.ac.kr (M.J.H.); jwkim@snu.ac.kr (J.K.); 4 Gachon Institute of Pharmaceutical Science, Gachon University; #191, Hambakmoe-ro, Yeonsu-gu, Incheon 21936, Korea; 5 Gachon Medical Research Institute, Gil Medical Center, Inchon 21565, Korea

**Keywords:** methylglyoxal, reactive oxygen species, human umbilical vein endothelial cells, *Peucedanum japonicum*, isosamidin, mitogen-activated protein kinases, advanced glycation end products, endothelial dysfunction

## Abstract

Methylglyoxal (MGO) is a highly reactive metabolite of glucose. Elevated levels of MGO induce the generation of reactive oxygen species (ROS) and cause cell death in endothelial cells. Vascular endothelial cell damage by ROS has been implicated in the progression of diabetic vascular complications, cardiovascular diseases, and atherosclerosis. In this study, the protective effect of isosamidin, isolated from *Peucedanum japonicum* roots, on MGO-induced apoptosis was investigated using human umbilical vein endothelial cells (HUVECs). Among the 20 compounds isolated from *P. japonicum*, isosamidin showed the highest effectiveness in inhibiting MGO-induced apoptosis of HUVECs. Pretreatment of HUVECs with isosamidin significantly prevented the generation of ROS and cell death induced by MGO. Isosamidin prevented MGO-induced apoptosis in HUVECs by downregulating the expression of Bax and upregulating the expression of Bcl-2. MGO treatment activated mitogen-activated protein kinases (MAPKs), such as p38, c-Jun N terminal kinase (JNK), and extracellular signal-regulated kinase (ERK). In contrast, pretreatment with isosamidin strongly inhibited the activation of p38 and JNK. Furthermore, isosamidin caused the breakdown of the crosslinks of the MGO-derived advanced glycation end products (AGEs). These findings suggest that isosamidin from *P. japonicum* may be used as a preventive agent against MGO-mediated endothelial dysfunction in diabetes. However, further study of the therapeutic potential of isosamidin on endothelial dysfunction needs to explored in vivo models.

## 1. Introduction

Hyperglycemic conditions, persisting over long periods of time, can lead to the formation of advanced glycation end products (AGEs). AGEs are formed endogenously by non-enzymatic glycation between the reducing sugars and the free amine groups of proteins. Increased concentrations of AGEs are well-recognized as mediators of diabetic complications, such as cataract generation, retinopathy, atherosclerosis, and nephropathy [1,2,3]. AGEs cause serious alterations in endothelial cells, such as mitochondrial dysfunction, cellular dysfunction, and, ultimately cell death [4]. The production of pro-inflammatory mediators is increased by the interaction between the AGEs and their receptors (RAGE); this in turn, triggers the generation of reactive oxygen species (ROS) [5,6,7]. Mitogen-activated protein kinases (MAPKs), such as c-Jun N terminal kinase (JNK), extracellular signal-regulated kinase (ERK), and p38, have been reported to be activated by AGEs [8].

Methylglyoxal (MGO) is a highly reactive metabolite of glucose; its accumulation is considered harmful. MGO is formed by both enzyme-catalyzed and non-enzymatic reactions [9]. Because of its strong oxidative and glycative properties, immediate detoxification of MGO, by the glyoxalase system is very important [10]. Recent studies reported that MGO levels can elevate in endothelial dysfunction as well as diabetes in vitro and in vivo model [11,12,13]. Cross-linking and glycation of proteins, caused by MGO, can lead to accelerated endothelial cell dysfunction [14,15,16]. Several studies reported that MGO-induced cytotoxicity is correlated with ROS production in endothelial cells [17,18]. Intracellular levels of ROS can be elevated by MGO and its modified proteins [19]. Further, increased MGO levels have been shown to regulate MAPKs in endothelial cells [20,21]. Therefore, endothelial dysfunction may be directly related to the carbonyl stress induced by MGO. Recently, many studies reported that several compounds from natural products can protect human umbilical vein endothelial cells (HUVECs) from MGO-mediated glucotoxicity [22,23].

Coumarins, a family of benzopyrones, have been associated with several beneficial effects on human health owing to their antioxidant, anti-inflammatory, and anti-fibrosis effects. Therefore, coumarins may ameliorate MGO-induced endothelial dysfunction and organ damage by scavenging ROS and downregulating inflammation and fibrosis. To identify the active molecules with MGO level-regulating properties, we screened *P. japonicum* for high levels of coumarins.

*P. japonicum*, a therapeutic plant of the family Umbelliferae, is found in southern and eastern Asia [24]. In Korea and Taiwan, *P. japonicum* roots have traditionally been used as folk medicine for the treatment of cold, cough, and neuralgic diseases. Previously, it has been reported that *P. japonicum* has anti-obesity [25,26], anti-diabetic [27], anti-adipogenic [25,27], antioxidant activity [28], and anti-inflammatory properties [29]. However, it is unclear whether the phytochemicals from *P. japonicum* roots can ameliorate endothelial dysfunction and, thereby, decrease apoptosis in vascular endothelial cells.

Thus, in the present study, we isolated 20 compounds from *P. japonicum* roots. We aimed to examine the anti-glycation and cytoprotective effects of the coumarins obtained from *P. japonicum* in HUVECs and elucidate the mechanism underlying their protective effects against MGO-induced glucotoxicity. We expect that our results will contribute to MGO-induced endothelial dysfunction in HUVECs.

## 2. Materials and Methods

### 2.1. Materials

*P. japonicum* roots were collected from Taean-gun in the Chungcheongnam-do province of South Korea in November, 2012 [24]. Twenty compounds were obtained from Seoul National University, Korea. MGO, aminoguanidine (AG), α-tubulin (cat no. T5168), and 2′,7′-dichlorofluorescein diacetate (DCF-DA) were purchased from Sigma (St. Louis, MO, USA). Bovine serum albumin (BSA; C0082-100) was obtained from RD tech (MOREBIO, Gyeonggi province, South Korea). EGM-2 medium was obtained from Lonza (Walkersville, MD, USA). Antibodies against p38 (cat no. 9212S), phospho-p38 (p-p38; cat no. 9211S), JNK (cat no. 9252S), phospho-JNK (p-JNK; cat no. 9251S), ERK (cat no. 9252S), and phospho-ERK (p-ERK; cat no. 9101S) were obtained from Cell Signaling Technology (Danvers, MA, USA). Bcl-2 (cat no. sc-492), Bax (cat no. sc-493), GLO-I (cat no. sc-67351), and Nrf2 (cat no. sc-365949) were purchased from Santa Cruz Biotechnology (Santa Cruz, CA, USA).

### 2.2. Extraction and Isolation

The extract of *P. japonicum* roots and its isolated twenty compounds were obtained and verified by Dr. Jinwoong Kim (Seoul National University). It was prepared as per the protocol of Kim et al. [24]. Firstly, the dried roots of *P. japonicum* (30.0 kg) were extracted with MeOH at room temperature (25 °C) in an ultrasonicator and filtered. Afterwards, the MeOH extract was partitioned with n-hexane, CHCl_3_, EtOAc, *n*-BuOH, and residual aqueous fractions. Kim et al. isolated twenty compounds from each subfraction according to the general analytical methods.

### 2.3. Cell Culture

HUVECs were purchased from the American Type Culture Collection (ATCC, Manassas, VA, USA; lot # 60319874) and maintained in EGM-2 medium, supplemented with 2% fetal bovine serum (FBS), at 37 °C in a humidified incubator containing 5% CO_2_. All cells used for the experiments were between passage number 5 and 8.

### 2.4. Cell Viability Analysis

The MTT (3-(4,5-dimethylthiazol-2-yl)-2,5-diphenyltetrazolium bromide) assay was used to evaluate cell viability. HUVECs were seeded at a density of 1.0 × 10^4^ cells/well in 96-well plates; they were then treated with the test compounds for 1 h, followed by treatment with MGO for 24 h. Subsequently, the cells were incubated for 24 h at 37° C. After incubation, the media containing the compounds was removed and the MTT solution was added; the cells were then incubated in a CO_2_ incubator for 2 h at 37 °C. After removing the media, 100 µL of dimethyl sulfoxide was added to each well. Subsequently, the absorbance was measured at 570 nm using a VERSA max microplate reader (Molecular Devices, CA, USA).

### 2.5. LDH Production Assay

The LDH production was evaluated using the Pierce LDH cytotoxicity assay kit (Thermo Scientific, Waltham, MA, USA) according to protocol with as per manufacture’s’ instructions. HUVECs were seeded at a density of 1.0 × 10^4^ cells/well in 96-well plates and incubated for 24 h, then treated with the isosamidin for 1 h, followed by treatment with MGO for 24 h at 37° C. After incubation, the conditioned medium (50 μL) from the treated plates was transferred to a 96-well plate. Next, 50 μL of the substrate mixture was added to the 96-well plate. The plates were mixed by placing the plate on a shaker for 30 min in the dark. The absorbance was evaluated at 490 nm and 680 nm using a VERSA max microplate reader (Molecular Devices, San Jose, CA, USA).

### 2.6. Cell Apoptosis Assay

The effect of isosamidin on MGO-induced apoptosis in the HUVECs was analyzed by flow cytometry (FACSCalibur flow cytometer; Becton Dickinson, San Jose, CA, USA) using an Annexin V apoptosis detection kit (Santa Cruz Biotechnology, Dallas, CA, USA). Briefly, the cells were seeded at a density of 5.0 × 10^5^ cells/well in a 6-well plate and incubated overnight at 37 °C. The cells were then treated with MGO (400 μM) and isosamidin (10 μM) for 24 h. Subsequently, the cells were harvested with trypsin-EDTA and washed with phosphate-buffered saline (PBS). The cells were then re-suspended in binding buffer with annexin V-FITC and propidium iodide (PI) and incubated at room temperature (25 °C) for 15 min in the dark. After incubation, 500 μL of PBS was added, and the percentage of apoptotic cells was analyzed by flow cytometry.

### 2.7. Measurement of Intracellular ROS

To determine the intracellular ROS scavenging activity of isosamidin, we used DCF-DA. HUVECs were seeded at a density of 1.0 × 10^5^ cells/well in a 12-well plate and incubated for 24 h at 37 °C. The cells were pretreated with isosamidin (10 μM) for 1 h, followed by treatment with MGO for 2 h. The cells were then washed with PBS and incubated with DCF-DA (10 μM) for 30 min at 37 °C. Subsequently, the distribution of DCF fluorescence was photographed using a JuLI live-cell imaging system (NanoEnTek, Seoul, Korea).

### 2.8. Measurement of AGE Breakdown

We performed the TNBSA (2,4,6-trinitrobenzene sulfonic acid) assay to evaluate the ability of the isosamidin to cause the breakdown of MGO-AGEs according to the method described by Furlani et al. [30]. We mixed a 1 mg/mL sample of preformed MGO-AGEs solution with 100 µM and 400 μM of isosamidin. This mixture was then incubated at 37 °C for 24 h. After incubation, 4% NaHCO_3_ (pH 8.5) and 0.1% TNBSA were added to each ep-tube for 2 h, at 37 °C; subsequently, the relative increase in the concentration of free amines was measured using a VERSA max microplate reader at 340 nm (Molecular Devices, CA, USA).

### 2.9. Western Blotting

To measure the protein the changes related to apoptosis and MAPKs, we performed Western blotting experiments in the HUVECs. After harvesting, the cells were lysed using PRO-PREP^TM^ (iNtRON Biotechnology, Seongnam, Korea), containing phosphatase and protease inhibitors. The concentration of total protein extracts was measured using the Bradford assay. Equal amounts of proteins were separated by SDS-PAGE and transferred to nitrocellulose membranes. The membranes were blocked for 1 h with 5% skim milk at room temperature and then incubated overnight with tubulin, p38, p-p38, JNK, p-JNK, ERK, p-ERK, Bcl-2, Bax, GLO-I, and Nrf2 primary antibodies at 4 °C. After 24 h, the membranes were conjugated for 1 h with secondary antibody at room temperature. additionally, washed and detected with TBST and ECL reagent (Millipore, Burlington, MA, USA). The bands were detected using a ChemiDoc XRS+ imaging system (Bio-Rad, Hercules, CA, USA).

### 2.10. Statistical Analysis

Statistical analysis was performed using one-way analysis of variance (ANOVA) in GraphPad Prism 5 (GraphPad Software, San Diego, CA, USA), followed by a Bonferroni’s post-hoc test. All data values were presented as the mean ± standard deviation (SD). A *p*-value < 0.05 was considered statistically significant.

## 3. Results

### 3.1. Effect of Isosamidin on MGO-Induced Cell Death in HUVECs

**Figure 1:**
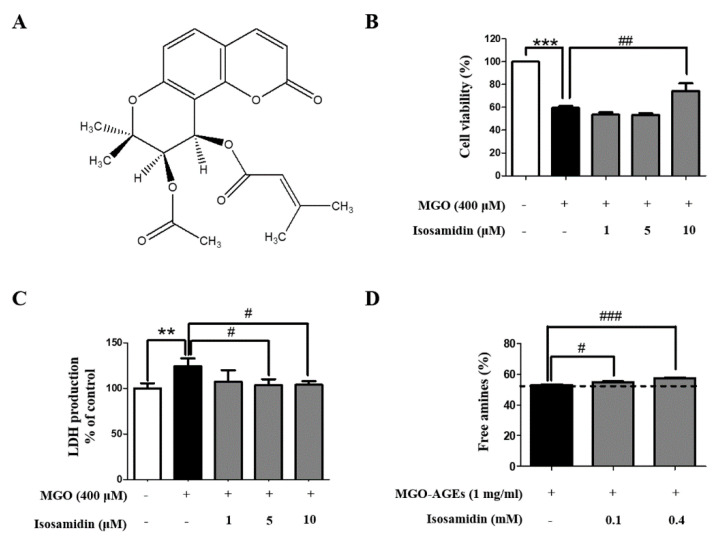
Effect of isosamidin isolated from *P. japonicum* roots on MGO-induced cytotoxicity in HUVECs. (**A**) Chemical structures of the compound. (**B**) Cell viability of the HUVECs treated with MGO and various concentrations of isosamidin (1, 5, and 10 μM) determined using the MTT assay. (**C**) MGO-induced LDH production in HUVECs measured using the LDH assay. (**D**) The MGO-AGE crosslinks breaking ability of isosamidin was evaluated by measuring the breaking of MGO-BSA using the TNBSA assay. The percent cell viability, LDH production, and free amines are presented as the mean ± SD of three independent experiments. (** *p* < 0.01, *** *p* < 0.001 vs. control, # *p* < 0.05, ## *p* < 0.01, ### *p* < 0.001 vs. MGO 400 μM, MGO-AGEs 1 mg/mL).

To select the *P. japonicum-*derived compounds with the most protective effects against MGO-induced toxicity, we performed cell viability screening of 20 compounds (10 μM each) using the MTT assay. As it exhibited the highest cytoprotective activity, isosamidin was selected (Table 1). The structure of the isosamidin is shown in Figure 1A. We investigated the effects of isosamidin on the viability of the HUVECs using the MTT assay. HUVECs were pretreated with 1, 5, and 10 μM of isosamidin for 1 h and then treated with 400 μM of MGO for 24 h. The MGO treatment markedly reduced the viability of the HUVECs, whereas pretreatment with isosamidin reversed this effect (Figure 1B). In fact, pretreatment with isosamidin with a concentration of 1–10 μM increased the cell viability in a dose-independent manner. However, the other compounds tested had no effect on the cell viability of the HUVECs (Table 1). In addition, MGO (400 μM) treatment significantly increased the LDH production in the HUVECs. LDH production significantly decreased in several concentrations (5 and 10 μM) of isosamidin (Figure 1C).

### 3.2. Effect of Isosamidin on the Crosslinks in AGEs

The anti-glycation activity of isosamidin was measured using the TNBSA assay. Isosamidin (100 μM and 400 μM) significantly exhibited breaking activity of the MGO-BSA-AGE crosslink. As shown in Figure 1D, isosamidin showed 4.64% crosslink breaking abilities at a concentration of 400 μM.

### 3.3. Effect of Isosamidin on MGO-Induced Apoptosis in HUVECs

**Figure 2:**
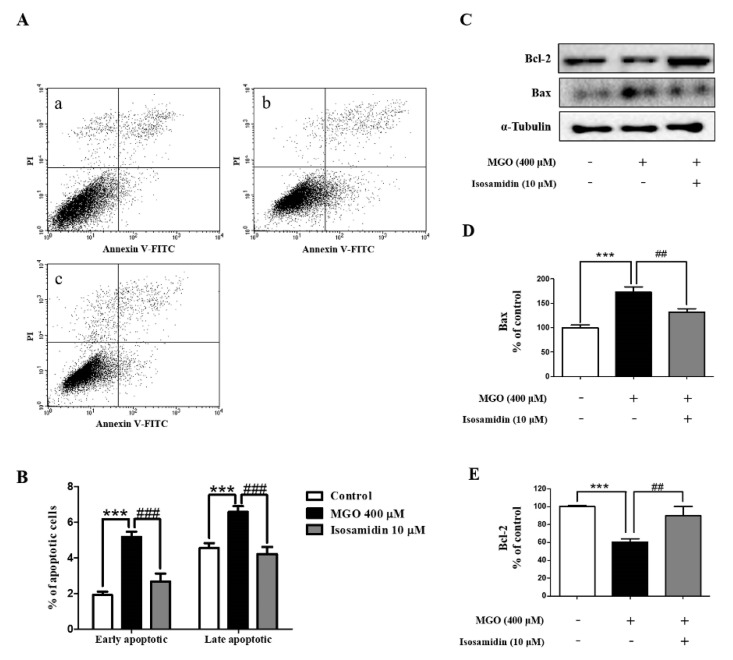
Effect of isosamidin on the MGO-induced apoptosis in HUVECs. (**A**) Annexin V-FITC and PI staining of MGO-stimulated HUVECs. Cells were pretreated without (−) or with (+) isosamidin for 1 h followed by treatment with MGO (400 μM). After 24 h, cells were harvested; cell apoptosis was analyzed by flow cytometry. (a) control; (b) 400 μM MGO; (c) MGO + isosamidin (10 μM). (**B**) Percentage of early and late apoptotic cells analyzed by flow cytometry. (*** *p* < 0.001 vs. control and ### *p* < 0.001 vs. MGO 400 μM treatment only) (**C**) Representative western blot of Bcl-2, Bax, and tubulin (internal control). (**D**) Relative expression band intensity level of Bax. (E) Relative expression band intensity level of Bcl-2. Bar values are presented as the mean ± SD of three independent experiments. (*** *p* < 0.001 vs. control, ## *p* < 0.01, ### *p* < 0.001 vs. MGO 400 μM).

To examine whether isosamidin could reduce the MGO-induced cell apoptosis, we performed FACS analysis, using annexin V-FITC and PI double staining. As shown in Figure 2A,B, MGO treatment led to an increase in the number of early and late apoptotic cells. However, the MGO-induced increase in early and late apoptotic activity was decreased after pretreatment with isosamidin.

### 3.4. Effect of Isosamidin on the Levels of Bax and Bcl-2

Western blotting was used to examine whether isosamidin could alter the MGO-induced expression of anti-apoptotic Bcl-2 and pro-apoptotic Bax proteins in HUVECs. As shown in Figure 2C–E, we observed that, compared to the control cells, MGO treatment resulted in a decrease in the expression of the Bcl-2 protein and an increase in the expression of the Bax protein. In contrast, treatment with isosamidin led to a decrease in the levels of Bax and an increase in those of Bcl-2.

### 3.5. Effect of Isosamidin on MGO-Induced ROS Generation

**Figure 3:**
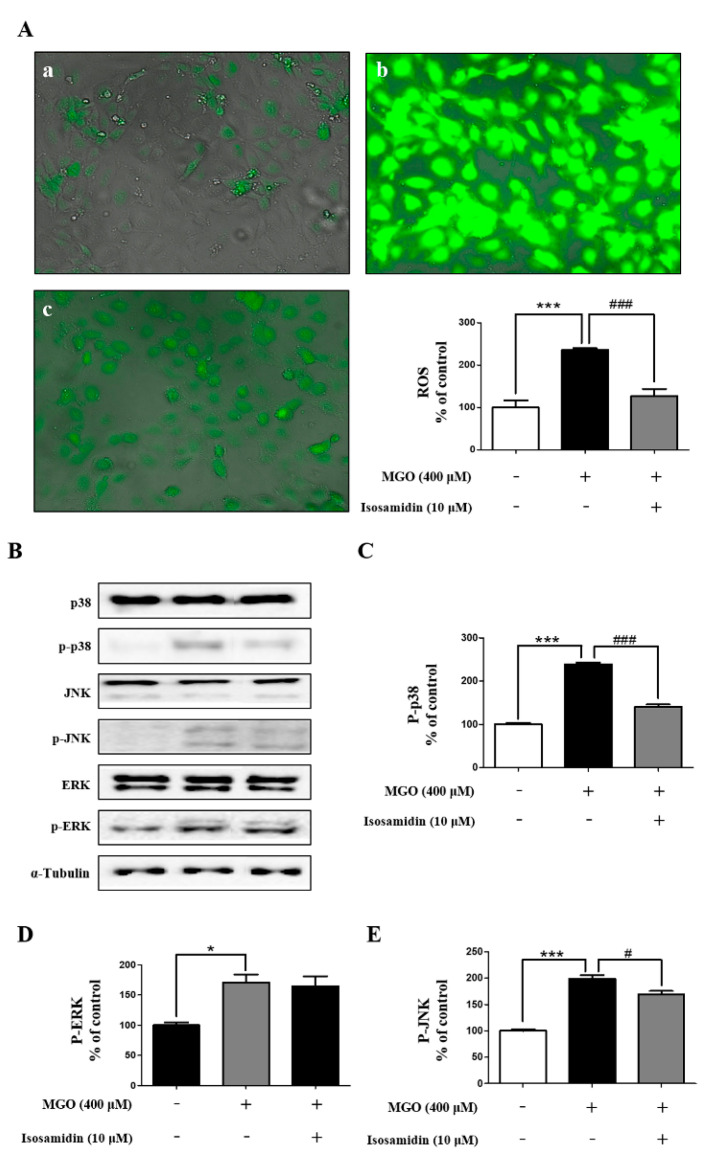
Effect of isosamidin on the MGO-induced MAPK signaling pathway activation via ROS generation in HUVECs. (**A**) HUVECs were pretreated with isosamidin for 1 h followed by treatment with MGO (400 μM) for 2 h. ROS generation was detected by staining with DCF-DA. (a) control; (b) MGO 400 μM; (c) MGO + isosamidin (10 μM). Western blots of total and phosphorylated forms of MAPKs. Cells were pretreated with (+) or without (−) isosamidin for 1 h followed by treatment with MGO 400 μM for 1 h. (**B**) Representative western blots of MAPKs. (**C**) The relative protein expression level of p-p38. (**D**) The relative protein expression level of p-JNK. (**E**) The relative protein expression level of p-ERK. Bar values are presented as the mean ± SD of three independent experiments. (* *p* < 0.05, *** *p* < 0.001 vs. control, # *p* < 0.05, ### *p* < 0.001 vs. MGO 400 μM).

The apoptosis of endothelial cells by intracellular ROS generation is a well-known phenomenon. Considering this, we further investigated whether the MGO-induced apoptosis in HUVECs is associated with increased ROS generation and whether isosamidin could decrease this ROS generation. As shown in Figure 3A, the intracellular level of ROS in the MGO-treated HUVECs increased significantly, whereas pretreatment with isosamidin markedly decreased the MGO-induced ROS generation.

### 3.6. Effect of Isosamidin on MAPK Activation

The MGO-induced apoptosis in endothelial cells is associated with phosphorylation-induced activation of MAPK. To assess the effect of isosamidin on MAPK signaling during MGO-induced apoptosis we examined the phosphorylation of p38, JNK, and ERK by Western blotting. As shown in Figure 3B–E, MGO treatment increased the phosphorylated forms of p38, JNK, and ERK, whereas the total protein levels of p38, JNK, and ERK remained unchanged. In contrast, pretreatment with isosamidin decreased the phosphorylation of p38 and JNK, whereas the phosphorylation protein levels of ERK remained unchanged.

### 3.7. Effect of Isosamidin on GLO-I and Nrf2 Expression

**Figure 4:**
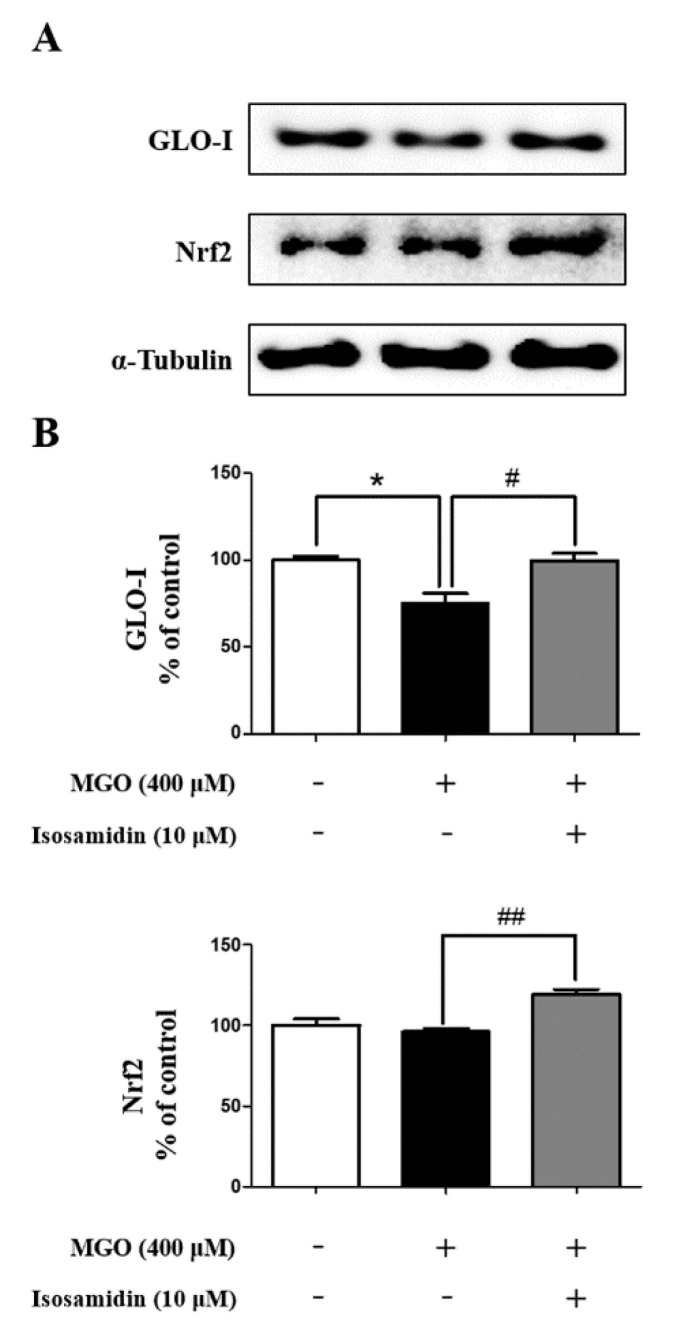
Effect of isosamidin on MGO-induced glyoxalase system related to protein expression in HUVECs. (**A**) Representative images displaying the protein expression of GLO-I and Nrf2. (**B**) The relative protein expression level of GLO-I and Nrf2. Bar values are presented as the mean ± SD of three independent experiments. (* *p* < 0.05 vs. control, # *p* < 0.05 and ## *p* <0.01 vs. MGO 400 μM).

The effect of the isosamidin on GLO-I and Nrf2 protein expression was measured using Western blotting in MGO-induced HUVECs. It showed that the levels of GLO-I decreased and Nrf2 unchanged in HUVECs following MGO treatment (Figure 4A). However, pretreatment with isosamidin increased the levels of GLO-I and Nrf2 in MGO-treated HUVECs (Figure 4B).

## 4. Discussion

MGO is a toxic dicarbonyl compound associated with diabetic vascular complications; it is known to trigger cellular injury and apoptosis in endothelial cells. Apoptosis of endothelial cells plays a key role in the development of atherosclerosis and cardiovascular disease. In this study, we confirmed that isosamidin, one of the coumarin compounds isolated from the roots of *P. japonicum*, could protect against MGO-induced apoptosis and oxidative damage. To evaluate the anti-apoptotic effects of the coumarins obtained from *P. japonicum*, we pretreated the cells with 20 compounds extracted from *P. japonicum* roots, at a concentration of 10 μM each for 1 h. In the present study, MGO treatment decreased the viability of HUVECs, thus indicating that MGO triggers direct cytotoxicity in HUVECs. We found that, out of all the compounds evaluated, only isosamidin had a potential protective effect on MGO-induced cell death (Table 1). However, pre-treatment of isosamidin concentration-independently (1, 10, and 100 μM) protected cell death in HUVECs (Figure S1). We also observed that isosamidin inhibited the cellular toxicity and lactate dehydrogenase (LDH) production (Figure 1C) observed in the MGO-induced apoptotic HUVECs. Resultantly, we selected 10 μM concentration of isosamidin for further mechanism study. In addition, annexin V-FITC/PI double staining demonstrated that MGO treatment increased apoptosis in HUVECs. Therefore, isosamidin protected the MGO-induced apoptotic cells (Figure 2A,B). We observed that isosamidin is effective in both the early and late stages of apoptosis. Hence, isosamidin appears to play a crucial role in the apoptosis induced by MGO in HUVECs.

Bcl-2 and Bax proteins, members of the Bcl-2 family, are involved in the modulation of cell apoptosis [31,32]. The induction of cell apoptosis can be determined by the alterations in the ratio of these two proteins. We found that exposure of HUVECs to MGO decreased the expression of Bcl-2 and increased the expression of Bax. However, pretreatment with isosamidin inhibited the MGO-induced apoptosis by decreasing the level of Bax and increasing the level of Bcl-2 (Figure 2C–E). Therefore, isosamidin might influence MGO-induced cell apoptosis via regulation of Bcl-2 and Bax levels in HUVECs.

MGO is a highly reactive metabolite that forms advanced glycation end products (AGEs) via various metabolic pathways, including fragmentation of triose phosphates during glycolysis, ketone body metabolism, threonine catabolism, and lipid peroxidation [33,34,35,36,37,38]. MGO reacts directly with the free sulfhydryl (SH) group or amino residues (lysine, arginine, and cysteine) on proteins to form AGEs that affect protein function [39,40]. The MGO-induced glycated protein (carboxyethyl-lysine; CEL) leads to the subsequent activation of the receptor of AGEs (RAGE), which then initiates vascular complications [41,42]. Previous studies have reported that MGO can induce endothelial dysfunction and apoptosis, mainly via generation of ROS and high levels of AGEs [43,44]. Therefore, we investigated whether isosamidin could increase the breakdown of the crosslinks in MGO-AGEs, which would imply that it could improve AGE-related vascular endothelial dysfunction. Resultantly, isosamidin showed anti-glycation effects that resulted in the breakdown of the crosslinks in the MGO-induced AGEs (Figure 1D) but it did not lead to a decrease in AGE formation (Figure S2); however, further studies using HPLC and LC-MS/MS needs to be conducted to explore this effect.

Several studies have suggested that intracellular ROS generation can be increased by MGO; thus, this may play an important role in AGE-RAGE formation [45,46,47]. Furthermore, Deshpande et al. reported the ROS-mediated proteolytic cleavage of procaspase-3 in the mitochondria of HUVECs [48]. Therefore, using DCF-DA, we investigated whether isosamidin could alter the MGO-induced generation of ROS. Our results showed that isosamidin pretreatment significantly reduced the MGO-induced generation of ROS (Figure 3A). Gupta et al. reported that the cell apoptosis induced by ROS generation was associated with the mitochondrial pathway and that Bcl-2 can regulate ROS signaling in the cells [49]. In another study, it was reported that the increase in Bax expression can upregulate intracellular ROS generation [50]. Therefore, this downregulation of the Bax/Bcl-2 ratio by isosamidin may be important for cell apoptosis, under the conditions of MGO-induced ROS generation in the HUVECs.

MAPK signaling is a key modulator of cell differentiation and cell apoptosis [51]. Many studies reported that phosphorylation of p38, JNK, and ERK leads to apoptosis [52,53,54]. Recently, several studies also reported that the activation of the MAPK family members, including JNK and p38, is associated with MGO-induced cytotoxicity [55,56]. In this study, we determined the effect of isosamidin on the MGO-induced inhibition of MAPK phosphorylation. We observed that pretreatment with isosamidin significantly inhibited the activation of p38 and JNK (Figure 3B–E) in the MGO-activated MAPK signaling pathway. The results of the present study demonstrated that the inhibition of apoptosis by isosamidin was accompanied by the inhibition of MAPK activation. These data suggested that isosamidin could modulate the MAPK signaling pathways in MGO-treated HUVECs.

Detoxification enzymes, such as glyoxalase-I (GLO-I), are associated with the activation of the nuclear factor erythroid 2-related factor 2 (Nrf2)/antioxidant response element (ARE) pathway [57,58]. Nrf2 is an important component of the transcription factor regulating the expression of genes containing an ARE responsible for protection against oxidative stress and glutathione recycling [58]. Therefore, the development of GLO-I inducers via the activation and binding of Nrf2 to the GLO-I functional ARE is a promising strategy. This upregulation of GLO-I and Nrf2 expression by isosamidin may be vital for the detoxification of MGO-induced glucotoxicity in HUVECs.

## 5. Conclusions

**Figure 5:**
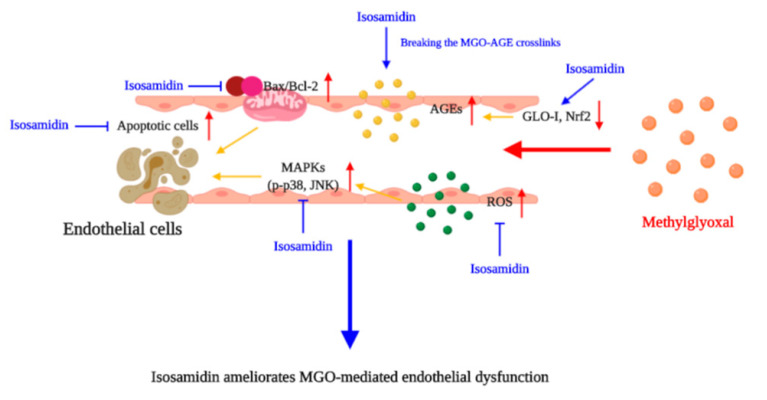
Hypothetical model of the molecular targets of isosamidin in the amelioration of MGO-induced apoptosis and glucotoxicity.

Isosamidin from the roots of *P. japonicum* showed cytoprotective effects in HUVECs by reducing the MGO-induced apoptosis by regulating the generation of ROS and the apoptotic signaling cascades. It was demonstrated that isosamidin holds great potential as a cytoprotective agent against MGO-induced cell toxicity. In addition, it also possesses anti-glycation and anti-apoptosis effects (Figure 5). These findings suggest that isosamidin from *P. japonicum* may play a protective role in MGO- and MGO-AGE-related endothelial dysfunction.

## Figures and Tables

**Table 1:** Effect of the compounds isolated from the roots of *P. japonicum* on methylglyoxal (MGO)-induced glucotoxicity in human umbilical vein endothelial cells (HUVECs).

Sample	Cell Viability (%)
MGO (400 μM)	100.00 ± 1.93
(3′S,4′S)-3′-acetyl-4′-angeloylkhellactone (pteryxin)	100.54 ± 2.21
(3′S,4′S)-3′,4′-disenecioylkhellactone	105.01 ± 2.54
(3′S,4′S)-3′-angeloyl-4′-senecioylkhellactone (calipteryxin)	105.47 ± 1.24
(3′R,4′R)-3′4′-diacetylkhellactone (qianhucoumarin D)	98.80 ± 7.85
(3′S,4′S)-3′,4′-diangeloylkhellactone (anomalin, praeruptorin B)	109.33 ± 1.92
(3′S,4′S)-3′-(2-methylbutyroyl)-4′-senecioylkhellactone	92.41 ± 3.18
(3′S,4′S)-4′-angeloyl-3′-(2-methylbutyroyl)khellactone	98.72 ± 4.83
(3′S,4′S)-4′-angeloyl-3′-senecioylkhellactone	101.66 ± 5.57
(3′S,4′S)-4′-isobutyroyl-3′-(2-methylbutyroyl)khellactone	91.43 ± 2.46
(3′S,4′S)-3′-isovaleroyl-4′-senecioylkhellactone	104.29 ± 2.32
(3′S,4′S)-4′(2-methylbutyroyl)-3′-senecioylkhellactone	97.11 ± 1.19
(3′S,4′S)-3′-acetyl-4′-isobutyroylkhellactone (hyuganin D)	96.83 ± 4.51
(3′S,4′S)-3′-acetyl-4′-senecioylkhellactone (isosamidin)	123.37 ± 2.77 ***
(3′S,4′S)-3′-acetyl-3′-isovaleroylkhellactone (suksdorfin, orymbocoumarin)	96.83 ± 2.01
(3′S,4′S)-3′-angeloyl-4′-(2-methylbutyroyl)khellactone (praeruptorin F)	102.87 ± 2.49
(3′S,4′S)-3′,4′-diisovaleroylkhellactone	95.65 ± 1.99
(3′S,4′S)-3′-isovaleroyl-4′-(2-methylbutyroyl)khellactone (praeruptorin H)	104.25 ± 2.94
(3′R)-O-senecioyllomatin	99.05 ± 1.50
Suberosin	90.41 ± 1.27
2′-hydroxyl-3′-senecioylvaginidiol	91.08 ± 3.18
Aminoguanidine (1 mM)	140.79 ± 3.29 ***

The percentage cell viability of each compound is presented as the mean ± SD of three independent experiments (*** *p* < 0.001 vs. MGO 400 μM).

## Supplementary Materials

The following are available online at https://www.mdpi.com/2076-3921/9/6/531/s1, Figure S1: Effect of isosamidin isolated from P. japonicum roots on MGO-induced glucotoxicity in HUVECs, Figure S2: Effect of isosamidin on MGO-induced glucotoxicity.

## Abbreviations

HUVECs	Human umbilical vein endothelial cells
MAPKs	Mitogen-activated protein kinases
JNK	c-Jun N terminal kinase
ERK	Extracellular signal-regulated kinase
AGEs	Advanced glycation end products
ROS	Reactive oxygen species
MGO	Methylglyoxal
DCF-DA	2′,7′-dichlorofluorescein diacetate
FBS	Fetal bovine serum
PBS	Phosphate-buffered saline
PI	Propidium iodide
MTT	3-(4,5-dimethylthiazol-2-yl)-2,5-diphenyltetrazolium bromide
TNBSA	2,4,6-trinitrobenzene sulfonic acid
LDH	Lactate dehydrogenase
CEL	Carboxyethyl-lysine
RAGE	Receptor of AGEs
GLO-I	Glyoxalase-I
Nrf2	Nuclear factor erythroid 2-related factor 2
ARE	Antioxidant response element
