# Supplementary material for: Isosamidin from Peucedanum japonicum Roots Prevents Methylglyoxal-Induced Glucotoxicity in Human Umbilical Vein Endothelial Cells via Suppression of ROS-Mediated Bax/Bcl-2
Source: Antioxidants (Basel). 2020 Jun 17;9(6):531. doi: 10.3390/antiox9060531 (PMC7346203; doi:10.3390/antiox9060531)
Supplement: Supplementary file 1 [file antioxidants-09-00531-s001.pdf]

A

B

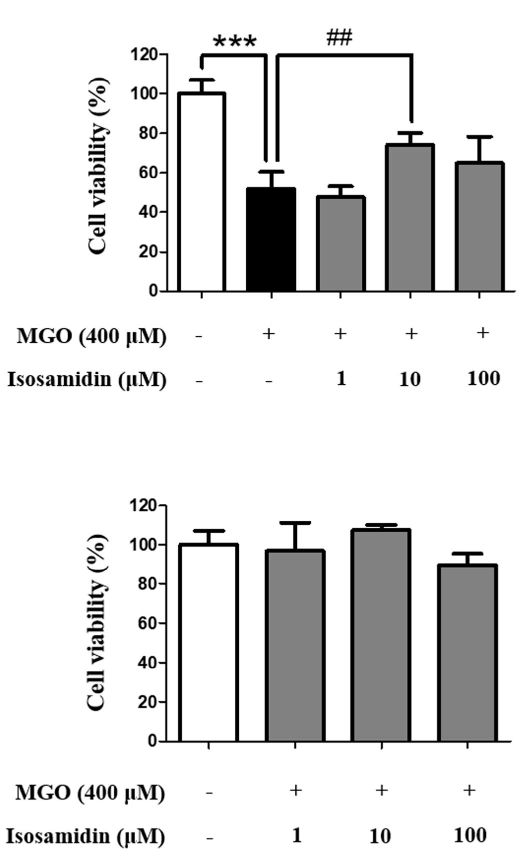

Supplementary Figure S1. Effect of isosamidin isolated from *P. japonicum* roots on MGO-induced glucotoxicity in HUVECs. (A) Cell viability of HUVECs treated with MGO (400 µM) and various concentrations (1, 10, and 100 µM) analyzed using the MTT assay. The percent cell viability is presented as the mean ± SD of three independent experiments. (***$p < 0.001$ vs. control, ##$p < 0.01$ vs. MGO 400 µM).

A

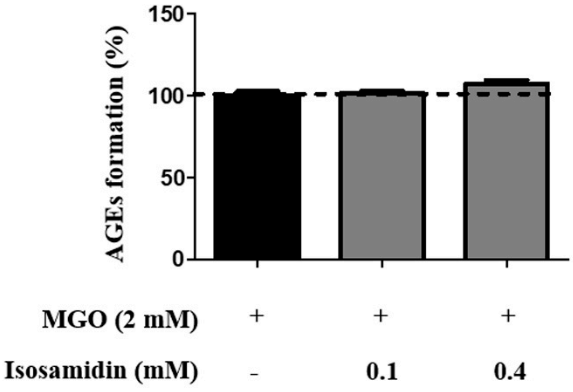

Supplementary Figure S2. Effect of isosamidin on MGO-induced glucotoxicity. (A) The AGEs formation inhibiting the ability of isosamidin was determined by measuring the inhibiting MGO-BSA-AGE using the AGEs formation assay. The percent AGEs formation is presented as the mean ± SD of three independent experiments.
